# Anchoring Mechanism for Capsule Endoscope: Mechanical Design, Fabrication and Experimental Evaluation

**DOI:** 10.3390/mi13122045

**Published:** 2022-11-22

**Authors:** Muhammad Rehan, Andrew G. Yeo, Muhammad Uzair Yousuf, Ebubekir Avci

**Affiliations:** 1 Department of Mechanical and Electrical Engineering, Massey University, Palmerston North 4410, New Zealand; 2 Electronic Engineering Department, Sir Syed University of Engineering & Technology, Karachi 75300, Pakistan; 3 Department of Mechanical Engineering, NED University of Engineering and Technology, Karachi 75270, Pakistan; 4 MacDiarmid Institute for Advanced Materials and Nanotechnology, Wellington 6140, New Zealand

**Keywords:** anchoring mechanism, robotic capsule, capsule endoscope, locomotion mechanism

## Abstract

Capsule endoscopes are widely used to diagnose gut-related problems, but they are passive in nature and cannot actively move inside the gut. This paper details the design process and development of an anchoring mechanism and actuation system to hold a capsule in place within the small intestine. The design centres around the mechanical structure of the anchor that makes use of compliant Sarrus linkage legs, which extend to make contact with the intestine, holding the capsule in place. Three variants with 2 legs, 3 legs and 4 legs of the anchoring mechanism were tested using a shape memory alloy spring actuator (5 mm × ϕ 3.4 mm). The experiments determine that all the variants can anchor at the target site and resist peristaltic forces of 346 mN. The proposed design is well suited for an intestine with a diameter of 19 mm. The proposed design allows the capsule endoscopes to anchor at the target site for a better and more thorough examination of the targeted region. The proposed anchoring mechanism has the potential to become a vital apparatus for clinicians to use with capsule endoscopes in the future.

## 1. Introduction

The human gut remained relatively unexplored until the early 19th century, as it mainly relied on observation using external diagnostic tools to determine potential gastrointestinal (GI) tract problems. The initial trials to measure gut parameters internally began in the late 1950s with an ingestible device, endoradiosonde, that was capable of measuring temperature, pressure and pH [1]. However, the major challenge of internal bleeding detection remained unsolved for a few decades. Sensors were experimented in similar-sized devices to detect gut lesions for monitoring internal bleeding but a viable solution was not found until the early 2000s, when a camera-based capsule was discovered [2]. This device, commonly known as a capsule endoscope or endoscopy, passes throughout the length of the gut, taking images along the journey. These images are then transferred wirelessly to a remote receiver to be examined by a clinician, aiding in the diagnosis of potential health problems, such as gut lesions or internal bleeding.

The capsule endoscope replaced the traditional tethered endoscopy that has limitations in accessing the entire gut, e.g., some parts of the small intestine. Furthermore, the tethered endoscope sometimes leads to gut perforation and bleeding, and the procedure itself is unpleasant and invasive for a patient [3]. After the proof of concept demonstration by Given Imaging with their M2A capsule [4], the first commercial capsule approved by the FDA, other companies also launched their capsules featuring similar or better diagnostic results [5]. All of the commercially available capsules mainly relied on natural peristaltic forces to push the capsule along the length of the gut. This resulted in the capsules having no control over their motion or orientation. While this is acceptable for a routine check-up of gut lesions, it does not provide any assistance in examining a specific location (target site) for a prolonged or detailed diagnosis. This limitation has opened the doors to further research. Various methods have been proposed to anchor the capsule at a target site to allow a clinician to perform diagnostics at the region of interest.

The anchoring mechanisms can be divided into two main categories, external control mechanisms (ECM) and internal control mechanisms (ICM). An ECM uses a permanent magnet inside the capsule that is manipulated by a magnetic field from outside the human or animal body. This is capble of holding (anchoring) the capsule at a target site [6]. The external magnetic field can be manipulated to precisely control the capsule’s motion inside the gut through the use of a large permanent magnet moved around the human or animal body with a robotic arm [7]. Alternatively, an array of electromagnets can be arranged to manipulate a magnetic field [8,9]. The ECM approach takes less space in the capsule, and its design is simple, as only a small magnet is placed inside the capsule. The external control approach has certain limitations. Firstly, the distance between the internal magnet inside the capsule and the external electromagnetic field generator needs to be very small; otherwise, the capsule loses its control. This greatly restricts the use of this method in clinical (in vivo) settings [9]. Secondly, the external control setup is expensive and requires additional technicians to operate the capsule, adding complexity to diagnostic procedures and creating an excessive burden on healthcare system [10]. Lastly, the overall gut structure is not stable. It is not firmly tied with the body, which makes the external control less effective for an anchoring mechanism.

The second approach to anchor the capsule at the target site is ICM. This utilises on-board mechanical components and micro-electro-mechanical system (MEMS)-based actuators [11]. The capsule carries the electronic actuator and an energy storage system, typically a battery, to induce motion that allows the capsule to anchor at the target site. This method does not require expensive external hardware and special operator assistance and can be automated to respond to environmental changes, e.g., pH, making them easier to use. A drawback to this method is the required space within the capsule needed to house the actuation system. This severely restricts the available working area for primary functionality, such as an on-board camera and battery [12,13]. Though this is a major design constraint in capsule development, through the improvements in energy storage [14] and micro-manufacturing processes [15], the limitations of space are less restrictive. Components are taking up less space, actuators are more energy efficient, and energy density in batteries is improving. These improvements have increased the feasibility of a self-contained robotic capsule system.

In this work, an anchoring mechanism is presented that can overcome the peristaltic forces of the small intestine to anchor at a target-site for detailed diagnosis of gut lesions. A low-profile anchoring design is proposed, providing a capsule room for onboard hardware to perform primary functions, such as endoscopy, drug delivery or sampling within the commercial capsule sizes. The anchoring mechanism is activated by a shape memory alloy (SMA) spring actuator (5 mm × ϕ 4 mm), which is powered by a high-drain current battery (10 mm × ϕ 10 mm). The anchoring mechanism can be activated by a wireless transceiver (10 mm × ϕ 10 mm) once the capsule reaches the target site. The main novelties of this work are detailed below:

1. The design and development of a low footprint anchoring mechanism that fit within a capsule endoscope and can anchor at the target site in the gut;
2. The design and selection of the custom-built SMA spring actuator to operate the anchoring mechanism;
3. The development of a miniaturised actuation system based on an SMA spring actuator by tackling the high-drain current requirements using a custom-built battery and a miniaturised wireless transceiver.

In this paper, an anchoring system with three variants (2 legs, 3 legs and 4 legs) is fabricated and rigorously tested on small intestine (ex vivo) of a pig. The anchoring systems are analysed to overcome the amount of peristaltic forces that are applied inside the small intestine in living conditions. The experiments determine that the proposed anchoring system can overcome the peristaltic forces to stay longer at the target site, hence allowing the clinicians to perform better diagnostics. The experimental results provided a proof of concept case for the in vivo testing that is the next stage of this project.

## 2. Materials and Methods

### 2.1. Mechanical Design of Anchoring Mechanism

**Figure 1:**
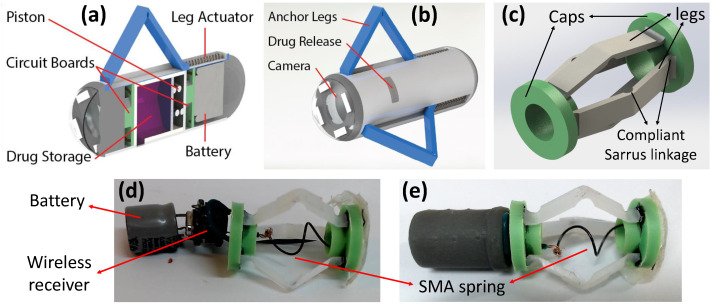
(**a**) Internal view and (**b**) external view of anchoring mechanism for capsule endoscope with simultaneous drug delivery. (**c**) Proposed anchoring mechanism design. Fabricated capsule prototype with (**d**) exploded view. (**e**) Assembled capsule.

The proposed anchoring system for the capsule endoscope with simultaneous drug delivery is shown in Figure 1a,b.

#### 2.1.1. Capsule Size

The physiological differences in the multiple organs that make up the GI tract have a major influence on the design of any device that will traverse it. The major limitation for this anchoring system is the size of the small intestine and the sphincter muscles that separate it from the stomach and large colon. Commercial capsules that have been granted FDA approval can be as large as 32.3 mm in length and 11.6 mm in diameter [16]. These dimensions became the targeted dimensions for the developed device in its unanchored state.

#### 2.1.2. Parameters of Small Intestine

**Figure 2:**
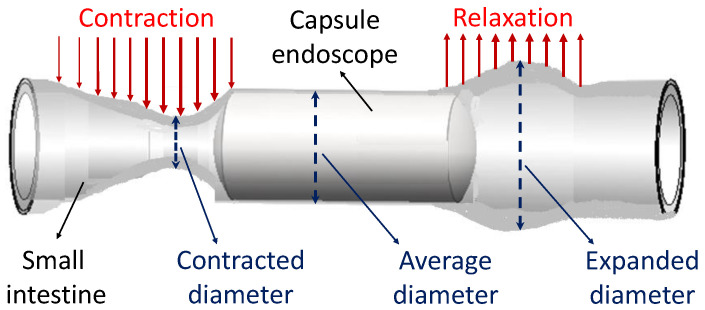
Conceptual model of a small intestine showing peristaltic forces on a capsule endoscope. The peristaltic cycle results in a range of diameters, which are illustrated and labelled for this study.

Anchoring is achieved by contracting the end caps of the anchoring mechanism that extends legs outside the capsule shell, as shown in Figure 1, which allows the capsule to resist the peristaltic forces. Therefore, it is critical to determine the lumen size of the small intestine, which is dynamic as it changes during the peristaltic cycle, as depicted in Figure 2. A study on 11 adult volunteers with no impairments showed a mean luminal diameter of 11.1 mm of the small intestine [17]. A separate study identified the mean amplification (between the contracted state and expanded state) of the small intestine to be 10.4 ± 4.53 mm [18]. From these studies, the upper bound of 19 mm diameter was used as the benchmark lumen size that the capsule must be capable of extending its legs to anchor inside the small intestine.

#### 2.1.3. Development of Anchoring Mechanism

**Figure 3:**
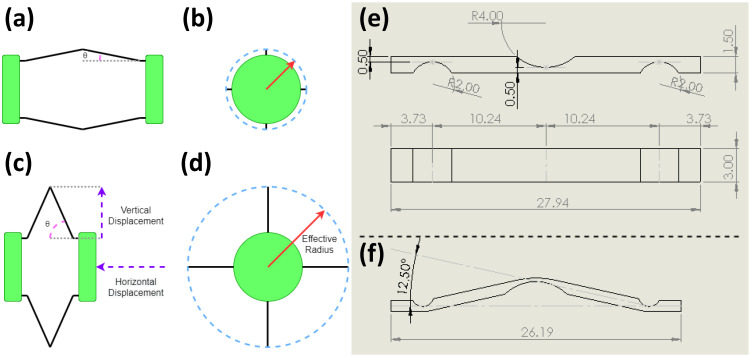
Design consideration of the anchoring mechanism. (**a**) Side view and (**b**) top view of the anchor in the resting state. (**c**) Side view and (**d**) top view of the anchor in the actuated state. The red arrow line in (**b**,**d**) indicates the effective radius of the contact points that interact with the intestinal wall. The green end caps are compressed together to extend the anchoring legs outside, shown in (**a**,**c**). Schematic leg design showing (**e**) Sarrus linkages. (**f**) Angle θ.

The end caps of the anchoring mechanism are contracted to extend the legs outside the capsule shell. The variable θ is used to identify the relationship between the bending of the legs and the displacement required (contraction) of the end caps, as shown in Figure 3a–d. These legs are a compliant Sarrus linkage, as illustrated in Figure 3e,f. The legs make contact with the intestinal wall, exerting a uniform force across all the contact points, allowing the capsule to anchor at the target site. The anchoring legs need to overcome the peristaltic forces that were measured earlier in one of our studies and determined to be in the range of 92 mN and 346 mN (average 226 mN) in the axial direction [19].

The structural design of the robot can be separated into two major parts, the structural end caps and the compliant legs, as shown in Figure 1c. The end caps act as a rigid support structure that hold a number of flexible legs in place. These legs are a compliant Sarrus linkage with the desired bending points of the leg thinner than the rest of the leg, as shown in Figure 3e,f. This enables the leg to deform at the intended position without the need for fabrication of multiple components to make up small hinges. To optimise the space used by the anchoring mechanism, three variants with 2 legs, 3 legs and 4 legs were fabricated and tested to determine the optimum solution that can resist the peristaltic forces while leaving the maximum space for the primary functions of the capsule (e.g., endoscopy, drug delivery). The end caps are a rigid disk intended to hold the legs in a symmetric circular pattern and ensure that, when actuated, the force is evenly spread across the legs. As functionality is added, one of the ends will become the main chassis for the design, and the second will become a slip ring that can translate along the principle axis of the capsule.

#### 2.1.4. Capsule Fabrication

The device was fabricated with both plastic milling and 3D printing of parts to the schematics in Figure 3e. The legs were cut from a 1.5 mm thick sheet of ultra-high-molecular-weight polyethylene (UHMWPE). The sheet was cut to the correct length of the legs, and then a 2 mm ball nosed cutter was run down the desired folding lines, reducing the thickness of the sheet to 0.5 mm at those points. The plastic was then cut every 4 mm to produce the individual legs. This process allowed for tolerance repeatability over many leg samples. The end caps were 3D printed with an ABS plastic (Prusa i3 MKS+, lime green EasyABS filament). The legs had a push fit with the designed slots in the end caps, but the bond was reinforced with Loctite 480 (Henkel).

The required actuation force to compress the end caps was calculated by Equation (1), and the amount of vertical displacement was determined using Equation (2)

(1) $$F_{a}=\frac{F_{p}}{tan(\theta )}$$

(2) Y=Lsin(θ)

where $F_{a}$ is the required actuation force, $F_{p}$ is the peristaltic force, θ is the bending angle of the anchor legs, *Y* is the vertical displacement, and *L* is the length of the leg. The peristaltic force $F_{p}$ considered in the theoretical calculation was 500 mN, which was slightly higher than the actual force of 346 mN to accommodate manufacturing tolerances [19]. The length of the leg *L* used in the prototype was 10.24 mm.

The legs were initially manufactured as a flat surface and then bent to a set angle, as shown in Figure 3f. This was due to the high theoretical forces that would work against the actuating force if the legs were flat. Doing this reduced the total distance of the leg extension between the initial state and the actuated state. From these theoretical calculations, as shown in Equations (1) and (2), it was determined that if the anchor could actuate the bending of the legs, adjusting θ from 12.5 degrees to 70 degrees, a displacement of 7.4 mm could be achieved without needing a large starting actuation force.

**Figure 4:**
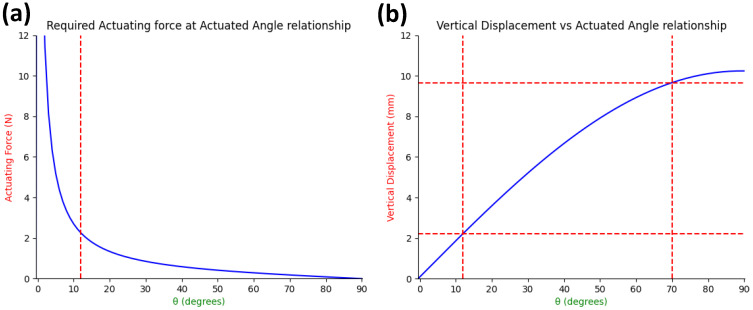
(**a**) The required actuating force to overcome the reactive force of a beam in relation to the angle the actuating force is applied to the beam. For the anchor’s case, an angle of 12.5 degrees (as indicated by the red line) was set to avoid the extreme peak that lower angles experience. (**b**) Theoretical vertical displacement the leg achieves when actuated from 0 to 90 degrees. The red lines indicate the chosen bounds of 12.5 to 70 degrees the capsule will actuate between, resulting in approximately 7.5 mm vertical displacement.

The theoretical forces were calculated using Equations (1) and (2) by viewing the system as a compressive force applied to the end of a beam. As the angle at which the actuating force is applied to the beam increases, the required force to begin actuation decreases exponentially, as shown in Figure 4a. An upper bound of 70 degrees was set because past this point, the extra vertical displacement achieved becomes negligible, as shown in Figure 4b. When actuated between 12.5 and 70 degrees, an effective radius of 12.9 mm in 2 and 4 leg variants of the device, and 12.4 mm in the 3 legged variant, could be achieved. An added benefit to setting θ to 12.5 degrees was ensuring that the legs would bend in the intended direction (outwards as opposed to inwards) when initially actuated.

#### 2.1.5. Biocompatibility and Strength of Capsule Material

The human gut is designed to protect itself from any harmful external objects and pathogens. The gut does not allow the interaction of external objects with the body, and external objects are naturally excreted from the gut along with faeces. However, it is critical that the material used for capsule endoscopy applications is bio-compatible to assure that the ingested materials does not harm the gut. In this study, the materials used for capsule fabrication are UHMWPE and ABS plastic which are bio-compatible that do not harm the human gut. However, the stomach environment in GI tract is a hostile environment for any external object, including capsule endoscopes, as this can damage the device. Therefore, some experiments were conducted to assure that the acidic environment of the stomach would not harm the proposed capsule.

The stomach contains hydrochloric acid (HCL) that maintains the overall pH at 1–3.5 and digestive juices (enzymes) that help in breaking the food down to be digested in the small intestine. The capsule endoscope is required to pass the stomach region before entering small intestine, and it must sustain itself in an acidic environment without any deterioration. Therefore, an in vitro gastric simulator was developed to replicate the stomach environment. The gastric juice was prepared using HCL and pepsin that was maintained at 1.5 pH, and the solution was kept circulating by a magnetic stirrer machine (VWR, US). Rectangular strips of 15 mm × 3 mm were prepared from both UHMWPE and ABS materials, which were then placed inside the in vitro gastric simulator for 1 day. In general, the capsule stays inside the stomach for up to 8 h, but the selected materials were tested for 24 h to ensure the safety of the host in case the capsule stays longer than usual transit time.

**Figure 5:**
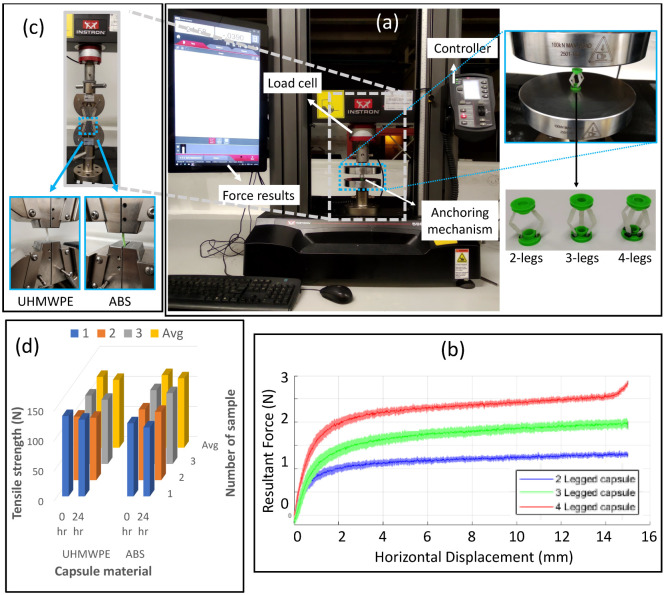
(**a**) Universal testing machine for measuring the compression force for each variant. (**b**) Force required to compress the end caps of the anchoring mechanism, measured by UTM. (**c**) UTM for measuring tensile strength of capsule materials (UHMWPE and ABS). (**d**) Tensile strength of material with and without exposure to stomach environment.

The tensile strength of the capsule material was tested to determine any potential deterioration in material strength at 0 h (without acid exposure) and 24 h (1 day of acid exposure) using a universal testing machine (UTM) (5967 Instron, US), as shown in Figure 5c. Three samples from each material were tested both with and without acid exposure to account for any manufacturing or testing variations, as shown in Figure 5d. Both materials showed nominal deterioration after 1 day acid exposure, which demonstrated that the proposed capsule will withstand the hostile stomach environment during in vivo testing.

### 2.2. Actuation System for Anchoring Mechanism

#### 2.2.1. Force Analysis of Fabricated Anchoring Mechanism

The force analysis was performed on the mechanical prototypes of the anchoring mechanisms to determine the required force for compressing the end caps from the resting (relaxed) state to the actuated (compressed) state, as shown in Figure 3a. The experimental setup and the fabricated variants of the anchoring prototypes are shown in Figure 5a. The compression tests were performed using a universal testing machine (UTM) (Instron 5967, US) that determined the required force for fully compressing the end caps, which in turn expands the legs to the maximum diameter. Three samples of each variants (2 legs, 3 legs, and 4 legs) were tested 3 times each by compressing the end caps with 15 mm displacement.

The amount of force required to produce the respective displacement by an anchoring mechanism is shown in Figure 5b. The displacement of 15 mm (fully compressed state) required an average force of 1.3 ± 0.041 N, 1.93 ± 0.088 N and 2.86 ± 0.043 N for the 2-legged, 3-legged and 4-legged anchors, respectively. A force of 2.58 ± 0.049 N was required to compress the 4-legged anchor to 14 mm displacement before the upturn occurred, as shown in the red color in Figure 5b. All the curves displayed similar characteristics, with the exception of the upturn at around 14.5 mm on the 4-legged variant. This upturn occurs with all three samples of the 4 legged variant, but it was not seen in the other two variants (2 legs and 3 legs). This happens because the 4-legged design creates a slight obstruction between the legs due to the narrow space (90${}^{{}^{\circ}}$ between each leg) when the structural end caps were fully squeezed to 15 mm displacement. The obstruction happens only after 14 mm, which can be reduced with design optimisation, but it was not required at this stage. Therefore the 4-legged variant is recommended to be used until 14 mm displacement to avoid any extra force after 14 mm. The lighter edge colour of each reading represents the upper and lower bounds of the measured readings, as each trial was performed three times (for each variant) to analyse the repeatability of the experiments. The experimental results determine that each additional leg increases the amount of required force, which can be seen in Figure 5b. This force analysis allowed us to develop an actuator that can offer the required output force to drive (compress) the anchoring mechanism at the target site.

#### 2.2.2. Design Requirements for Actuator Selection

A variety of actuators (motor, solenoid and SMA) were considered for the proposed anchoring mechanism design. The design parameters, such as the size, number of components, ease of use, power required, and force generated in each method, were considered [19,20]. Based on initial trials, a SMA spring actuator is considered feasible and optimised based on the design requirements. SMA is a versatile material that can be deformed and then returned to its “memory state” when it is subject to a temperature change. This transformation occurs because the alloy is changed between its martensite and austenite phases and their corresponding crystalline structures [21]. The alloy itself has its phase transition temperature engineered so it can occur at a manageable temperature. This results in a single component that can take the shape of a coil spring that changes its overall length [20].

#### 2.2.3. Design of SMA Spring Actuator

The actuation mechanism for the capsule endoscope needs to push or pull the end caps of the anchoring mechanism that will expand the anchoring legs to resist the peristaltic forces at the target site. The linear motion can be generated by an SMA spring actuator that can push or pull the anchoring mechanism when activated and returns to its original position once deactivated. The SMA spring actuator deflection (δ) can be defined as

(3) $$\delta =F\frac{8D^{3}n}{Gd^{4}}$$

where *F* is the load, *G* is the modulus of the rigidity, *d* is the diameter of the wire (wire size), *D* is the mean diameter of the spring and *n* is the number of turns in the spring.

**Figure 6:**
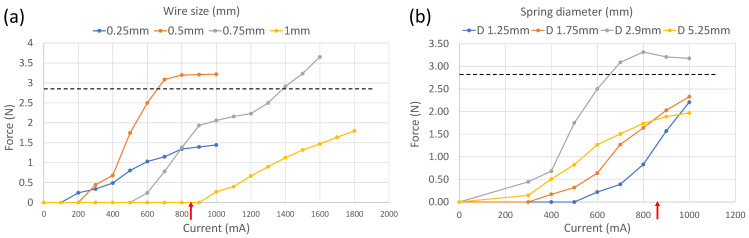
(**a**) Force measurement of different wire sizes at varying currents. (**b**) Force measurements of different spring diameters with 0.5 mm wire size at varying currents. The red arrow on x-axis indicates the amount of drain current supplied by the selected battery. The black line on y-axis indicates the peristaltic forces the SMA spring actuator needs to overcome to anchor at target site.

The spring wire size (*d*) is a key element in spring design and is directly proportional to the output load (force), as shown in Equation (3), but this also means that a higher amount of energy will be required to power (actuate) the SMA spring. To determine the optimum wire size, four wire diameters (0.25 mm, 0.5 mm, 0.75 mm and 1 mm) were tested on varying amount of currents, as shown in Figure 6a. The experimental results show that the higher wire size results in a higher output force, but this can only be possible with a high-drain current battery. The capsule endoscope can carry a limited battery size, and the battery used in our prototype can drain up to 850 mN current. Another design constraint is the amount of output force required. The SMA spring actuator needs to produce 1.3 N, 1.93 N and 2.86 N output forces for 2-legged, 3-legged and 4-legged anchoring mechanisms, respectively. Considering the highest value, an SMA spring actuator that can apply more than 2.86 N force is required to be utilised with any variant of the anchoring mechanism. Therefore, 0.75 mm and 1 mm wire sizes cannot apply enough output force when restricted to an 850 mA drain current, while the 0.25 mm wire size cannot apply the required force at all. Hence, the 0.5 mm wire size fits for both constraints, i.e., the drain current limits of the battery and output force requirements.

Another key variable in spring design is the spring diameter. Once the 0.5 mm wire size was selected, four different springs with various diameters were tested, as shown in Figure 6b, to analyse the impact on output force so the optimum wire diameter can be selected. The springs with mean diameters of 1.25 mm and 1.75 mm apply less than the desired output force, as the internal diameter was too small for the spring and restricted the elongation, even at higher drain current values. Similarly, the spring with diameter of 5.25 mm was too big for the 0.5 mm wire size, and the output force remained below 2 N (less than desired). The only spring that fulfilled the output force requirements was the spring with a mean diameter of 2.9 mm, which applied more than 3 N output force at the 700 mA and 800 mA currents. It was selected as the optimum candidate for the proposed anchoring mechanism.

#### 2.2.4. Development of Actuation System

**Figure 7:**
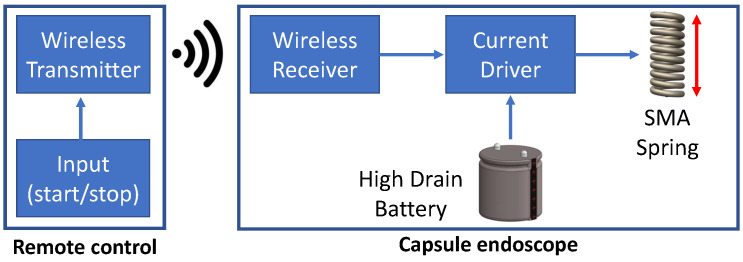
Actuation mechanism for capsule endoscope.

The actuation system consisting of an SMA spring actuator (Kellogg’s Research Lab, Hudson, OH, USA), a battery (NSC1010, Huahui New Energy, Yiyang, China) and a wireless transceiver (Shenzhen Anntem Technology Co. Ltd., Shenzhen, China) was developed to remotely trigger the anchoring mechanism when needed, as shown in Figure 7. The wireless transmitter works at 433 MHz frequency and can activate the capsule from within 5 m distance, which is perfect for capsule endoscope applications. Once the capsule is triggered from the remote control, the wireless receiver passes the current to the SMA spring through the high-drain battery. The SMA spring contracts (decreases in length) due to the change in heat that is produced by the flow of electrons through the spring wire (Joule heating). This contraction is utilised by the anchoring mechanism to compress the end caps and expand the anchoring legs to anchor at the target site. Please see Supplementary Materials for more information. The temperature difference between the expansion and compression states of the SMA spring is due to the hysteresis effect between the austenite and martensite states of the SMA material, which is explained in our previous work [22]. The capsule endoscope can be visualised using imaging devices, e.g., X-ray or ultrasound, and the anchoring mechanism can be activated wirelessly when the capsule reaches the target site.

#### 2.2.5. Simulator for Anchoring Trials

**Figure 8:**
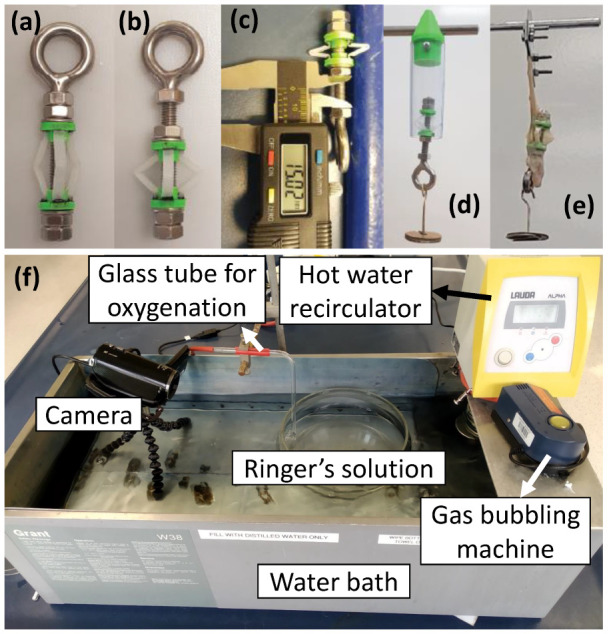
(**a**–**e**) Structural support to simulate compression of end caps for the anchoring mechanism. (**a**) Anchor mounted on support. (**b**) Anchor compressed by adjusting the support. (**c**) Fully compressed anchor with 15 mm displacement. Experimental setup for preliminary trials using (**d**) a vinyl tube and (**e**) ex vivo intestine. (**f**) In vitro experimental setup for maintaining the peristaltic movements of a freshly dissected intestine.

A structural support was developed that can compress the end caps of the anchoring mechanism to analyse the anchoring mechanism. An eye bolt was used along the principle axis on a 6 mm stainless steel shaft that secured the anchor in place with nuts on either side, as shown in Figure 8a. One nut could be tightened to compress the end caps of the anchor, as shown in Figure 8b. The anchoring mechanism was compressed to 15 mm (fully extended), as shown in Figure 8c, and then the preliminary trials were conducted on a transparent vinyl tube and an ex vivo small intestine as shown in Figure 8d,e respectively. The support and anchors weighed 28 g altogether, and the hook weighed 2 g.

#### 2.2.6. Living Intestinal Trials

A special experimental setup, shown in Figure 8f, that can keep an intestine alive for 6 h was utilised to test the anchoring mechanism [23]. This setup maintains the physiological parameters such as pH and temperature and provides the nutrients and oxygen required by an intestine to maintain its peristaltic movement. This experimental setup is the nearest proxy for an in vivo environment in terms of maintaining the peristaltic movements and has widely been used for nutritional and drug absorption studies [24].

## 3. Results and Discussion

### 3.1. Anchoring Trials on a Simulator

The anchoring prototypes with three variants, 2 legs, 3 legs and 4 legs, were fabricated and subjected to an anchoring simulation to determine the optimum number of legs for anchoring inside the small intestine. The prototypes were tested in two separate environments to rigorously test the three variants of the anchoring prototypes. First, they were tested in a transparent vinyl tube to observe the performance of the anchor on a smooth surface. Second, they were tested on ex vivo small intestines of a pig to validate the performance under an actual environment. A structural support was used to deploy the anchors (compressed state) and ensure the actuated distance remained the same throughout the test. A hanging weight was used to exert an axial load on the anchor to simulate the force induced by the peristaltic motion, as shown in Figure 8d,e. The peristaltic force that the anchor need to overcome was 346 mN in the axial (longitudinal) dimension [19], so this was used as the benchmark.

The anchors were first tested in a 19 mm (inner) diameter transparent vinyl tube, as shown in Figure 8d. The anchors were compressed by 6 mm, which increased the effective radius by 5 mm, and then left to hang at their body weight (294 mN) for 30 s. A load of 10 g (98 mN) was added after every 30 s interval until 100 g (980 mN) weight was achieved. After reaching the 100 g (980 mN) weight, an addition of 50 g (490 mN) weights were made after every 30 s up to 300 g (2940 mN). At 35 g (343 mN) weight, the anchor was experiencing approximately the maximum force it is expected to experience within a human intestine, but the anchors were tested to a 10-fold higher weight to determine their failure point. All variants of the anchors achieved holding 300 g (2940 mN) without slippage.

### 3.2. Anchoring Trials on Ex-Vivo Intestine

The anchors were tested on the small intestines of a pig, which were sourced from a local butcher. The 100 mm sections were cut from the duodenum (beginning region), jejunum (middle region) and ileum (ending region) of a small intestine for ex vivo trials. The approximate diameters of the three sections were 16 mm, 19 mm and 21 mm. The intestine with a 19 mm diameter was the benchmark size for the anchor, as the design was mainly prepared for the 19 mm lumen (diameter)-sized intestine.

The anchors were first placed into the 16 mm diameter section of the intestine and compressed by 6 mm, which increased the effective radius by 5 mm. The free end of the intestine was clamped between 2 stainless steel plates that were then hung from the test stand, as shown in Figure 8e. A similar procedure was repeated in terms of the addition of weights, as conducted for the vinyl tube experiments (Section 3.1). Three prototypes of each variant (2 legs, 3 legs and 4 legs) were tested, and the results are shown in Table 1. Similar experiments were repeated for the 19 mm and 21 mm diameters of intestines, but the end caps were compressed by 12 mm in these trials, which increased the effective radius by 7.5 mm.

All the variants tested in the vinyl tube and the 16 mm small intestine anchored successfully in all the trials for a 2940 mN load, which is 8.5 times the expected peristaltic forces (346 mN), without any failure point [19]. Therefore, it can be inferred that the proposed anchoring mechanism with any number of legs (2, 3 or 4) can successfully anchor inside a small intestine considering that the diameter is 16 mm or less. Secondly, the success in the intestine with a 19 mm diameter is directly proportional to the number of legs, as the average resistant forces by the anchoring mechanisms were 490 mN, 719 mN and 784 mN for the 2-, 3- and 4-legged anchors, respectively. Still, all the variants can successfully anchor inside the small intestine with a diameter of 19 mm, as each trial resulted in holding force of more than 346 mN. Lastly, none of the prototypes were able to be anchored inside the small intestine with a diameter of 21 mm; hence, anchoring with proposed design is not possible for diameters at or above 21 mm.

### 3.3. Anchoring Trials on Living Intestine

Freshly dissected intestines were sourced from a local butcher and placed inside the experimental setup, as shown in Figure 8f, after being cut into 300 mm sections. The capsule prototype with the 3-legged anchoring mechanism and actuation system were placed inside the small intestine. Once the small intestine retained the peristaltic forces, the capsule was activated using a remote control and observed for any movement. The capsule did not move by any observable distance. It is considered that the anchoring mechanism has the capability to withstand the peristaltic forces and anchor at the target site.

## 4. Discussion

The results show that the proposed anchoring mechanism has the potential to anchor inside a small intestine that is up to 19 mm in diameter with a success rate of 100%. However, if the intestinal diameter is more than and equal to 21 mm, than the anchoring legs cannot hold the intestinal wall to stick at the target site. This happens because the stretched legs of the anchor can firmly hold the intestinal wall with a 19 mm diameter but not with diameters larger than this. Considering that the average diameter of the small intestine is around 19 mm, the proposed design is feasible for anchoring inside the small intestinal region. For larger sizes of small intestines and for the colon region, the length of the legs can be increased, which will result in a wider stretch in the horizontal direction, resulting in generating resistance for higher diameter sizes.

The bio-compatibility of the materials used to fabricate the proposed prototype was tested for material strength after exposing the materials to a gastric simulator for 1 day. This allowed us to consider the safety of both the host and the device in this proof-of-concept design. In the future, a gut simulator that replicates the entire gut environment will be used to determine any potential failure points for the proposed prototype to fully understand the safety concerns before in vivo trials [22]. Furthermore, an enteric coating outside the capsule shell can be used to further protect the capsule from an acidic environment. The enteric coating remains intact in the stomach and dissolves in the small intestine region by reacting to the intestinal fluid [25].

The proposed design is based on squeezing the structural end caps through an actuation system, which stretches the legs outside the capsule shell to anchor at the target site. The legs are bent in the opposite direction of the capsule core by 12.5${}^{{}^{\circ}}$ to reduce the high actuation force and assure that the legs are stretched outside. The alignment of both end caps is critical for smooth operation, which was assured through manual alignment methods during fabrication. In the future, the capsule will include a central core that will hold the battery unit and camera module. This will assure the alignment between the two end caps for the smooth movement of the anchoring mechanism.

The proposed anchoring mechanism is well-suited for existing capsule endoscopes considering its low-footprint design. The internal space inside the proposed anchoring design can be utilised to simultaneously deliver a drug at the target site, as shown in Figure 1a,b, or used to secure a gut sample. The existing design is a proof-of-concept for the anchoring mechanism. In the future, it will be implemented within a robotic capsule, allowing it to perform additional functions, such as drug delivery, sampling, or prolonged observation at a desired target site.

## 5. Conclusions

A low-profile footprint anchoring mechanism is designed and developed in this work that can overcome the peristaltic forces of the small intestine to anchor at a target site for the detailed diagnosis of gut lesions. The fabricated anchoring mechanism prototype occupies a small footprint that can be incorporated within capsule endoscopes. These capsules could potentially include simultaneous functions such as drug delivery or sampling. The anchoring mechanism is activated by an actuation system that is comprised of an SMA spring actuator, which is powered by a high-drain current battery. The anchors can be deployed by a wireless transceiver once the capsule reaches a target site. The anchoring mechanisms with three variants (2 legs, 3 legs and 4 legs) were fabricated and rigorously tested, initially on a simulator, then within the ex vivo and living small intestines of a pig. The experiments determined that the proposed anchoring system can overcome the peristaltic forces, allowing a capsule to stay longer at a target site, and in turn aiding clinicians in performing better diagnostics. The experimental results provided a proof-of-concept case for the in vivo testing that is the next stage of this project.

## Figures and Tables

**Table 1:** Anchoring force measurement inside vinyl tube and intestine.

		Anchoring Force (mN)								
	**Diameter**	**2-legs**			**3-legs**			**4-legs**		
Tube	19 mm	2940	2940	2940	2940	2940	2940	2940	2940	2940
Intestine	16 mm	2940	2940	2940	2940	2940	2940	2940	2940	2940
	19 mm	392	588	490	588	882	686	784	882	686
	21 mm	0	0	0	0	0	0	0	0	0

## Data Availability

Not applicable.

## Supplementary Materials

The following supporting information can be downloaded at: https://www.mdpi.com/article/10.3390/mi13122045/s1.

## Abbreviations

The following abbreviations are used in this manuscript:

GI tract	Gastrointestinal tract
UHMWPE	Ultra-high-molecular-weight Polyethylene
SMA	Shape memory alloy
